# Validation of a novel multibiomarker test to assess rheumatoid arthritis disease activity

**DOI:** 10.1002/acr.21767

**Published:** 2012-11-28

**Authors:** Jeffrey R Curtis, Annette H van der Helm-van Mil, Rachel Knevel, Tom W Huizinga, Douglas J Haney, Yijing Shen, Saroja Ramanujan, Guy Cavet, Michael Centola, Lyndal K Hesterberg, David Chernoff, Kerri Ford, Nancy A Shadick, Max Hamburger, Roy Fleischmann, Edward Keystone, Michael E Weinblatt

**Affiliations:** 1 University of Alabama at BirminghamLeiden, The Netherlands; 2 Leiden University Medical CenterLeiden, The Netherlands; 3 Crescendo BioscienceSouth San Francisco, California; 4 Oklahoma Medical Research FoundationOklahoma City; 5 Brigham and Women's HospitalBoston, Massachusetts; 6 Rheumatology Associates of Long IslandMelville, New York; 7 University of Texas Southwestern Medical CenterDallas; 8 Mount Sinai Hospital, University of TorontoToronto, Ontario, Canada

## Abstract

*Objective* Quantitative assessment of disease activity in rheumatoid arthritis (RA) is important for patient management, and additional objective information may aid rheumatologists in clinical decision making. We validated a recently developed multibiomarker disease activity (MBDA) test relative to clinical disease activity in diverse RA cohorts.

*Methods* Serum samples were obtained from the Index for Rheumatoid Arthritis Measurement, Brigham and Women's Hospital Rheumatoid Arthritis Sequential Study, and Leiden Early Arthritis Clinic cohorts. Levels of 12 biomarkers were measured and combined according to a prespecified algorithm to generate the composite MBDA score. The relationship of the MBDA score to clinical disease activity was characterized separately in seropositive and seronegative patients using Pearson's correlations and the area under the receiver operating characteristic curve (AUROC) to discriminate between patients with low and moderate/high disease activity. Associations between changes in MBDA score and clinical responses 6–12 weeks after initiation of anti–tumor necrosis factor or methotrexate treatment were evaluated by the AUROC.

*Results* The MBDA score was significantly associated with the Disease Activity Score in 28 joints using the C-reactive protein level (DAS28-CRP) in both seropositive (AUROC 0.77, *P* < 0.001) and seronegative (AUROC 0.70, *P* < 0.001) patients. In subgroups based on age, sex, body mass index, and treatment, the MBDA score was associated with the DAS28-CRP (*P* < 0.05) in all seropositive and most seronegative subgroups. Changes in the MBDA score at 6–12 weeks could discriminate both American College of Rheumatology criteria for 50% improvement responses (*P* = 0.03) and DAS28-CRP improvement (*P* = 0.002). Changes in the MBDA score at 2 weeks were also associated with subsequent DAS28-CRP response (*P* = 0.02).

*Conclusion* Our findings establish the criterion and discriminant validity of a novel multibiomarker test as an objective measure of RA disease activity to aid in the management of RA in patients with this condition.

## INTRODUCTION

Measurement of disease activity has become an important component of rheumatoid arthritis (RA) management. Quantitative measurement of RA disease activity is recommended by the American College of Rheumatology (ACR) (1), the European League Against Rheumatism (EULAR) (2), and the Treat to Target task force (3). It has been included in the Physician Quality Reporting System (4) and is central to the evaluation of new RA treatments. Tight control strategies that incorporate regular measures of disease activity have been shown to improve patient outcomes, with greater reduction in disease activity, increased rates of remission, and improved radiographic outcomes (5–7).

Several disease activity indices based on different clinical, laboratory, and physical measures have been introduced. Most of these, including the Disease Activity Score (DAS) (8), the modified DAS in 28 joints (DAS28) (9), the Simplified Disease Activity Index (SDAI) (10), the Clinical Disease Activity Index (CDAI) (11), and the Routine Assessment of Patient Index Data 3 (RAPID3) (12, 13), rely on either quantitative joint counts, patient-reported outcomes (PROs), or both. Joint counts and PROs are critically important in patient assessment, but they exhibit significant intraobserver and interobserver variability and can be influenced by cumulative damage and conditions other than RA (e.g., fibromyalgia and osteoarthritis) (14–16). Additional objective information about underlying disease processes may augment clinical assessment.

Laboratory tests represent one approach to such additional information, and markers such as the erythrocyte sedimentation rate (ESR) and C-reactive protein (CRP) level have been incorporated into disease activity assessment in patients with RA (17, 18). However, both the ESR and CRP are nonspecific markers of inflammation and also can be increased by aging, anemia, and the presence of immunoglobulins, including rheumatoid factor (RF) (19–21). Furthermore, normal levels of these markers are seen in some RA patients with active disease (22), possibly in part due to patient genetics (19–21). Therefore, these markers may not be helpful in all RA patients.

We hypothesized that a multibiomarker test measuring diverse biologic pathways involved in RA could provide clinicians with a robust and objective measure of RA disease activity. Such a test could be used in conjunction with clinical examination and PROs in routine clinical practice and might prove particularly useful in circumstances where clinical assessment can be challenging or confounded, such as for patients with comorbidities. In previous work, we developed an algorithm that combines the levels of 12 serum biomarkers (interleukin-6 [IL-6], tumor necrosis factor receptor type I [TNFRI], vascular cell adhesion molecule 1 [VCAM-1], epidermal growth factor [EGF], vascular endothelial growth factor A [VEGF-A], YKL-40, matrix metalloproteinase 1 [MMP-1], MMP-3, CRP, serum amyloid A [SAA], leptin, and resistin) to generate a multibiomarker disease activity (MBDA) score between 1 and 100 (Vectra DA, Crescendo Bioscience) (23).

In the present study, we sought to establish the criterion and discriminant validity of the MBDA score as a measure of disease activity in patients with RA. The 5 aspects of validity previously proposed for the qualification of outcome measures in clinical trials in RA (24, 25) include: 1) criterion validity, the ability of a test to measure disease activity; 2) discriminant validity, sensitivity to changes in disease activity; 3) face validity, credibility and relevance; 4) content validity, comprehensiveness in measuring components of health status; and 5) construct validity, relationship to outcomes such as damage and disability. We established criterion validity of the MBDA test by demonstrating a significant association with the DAS28-CRP in an independent sample of patients with RA who had not been evaluated previously during the development of the test. The DAS28-CRP was chosen as the reference measure because it has been validated against the DAS28-ESR (26), is correlated with other clinically relevant RA outcomes in clinical trials (9) and, unlike the ESR, the CRP measurement can be standardized in archived samples from multiple centers. Furthermore, to establish the discriminant validity of the MBDA score, we evaluated whether changes in the MBDA score were associated with changes in the DAS28-CRP and with clinical responses in patients initiating methotrexate (MTX) or anti–tumor necrosis factor α (anti-TNFα) therapy. Finally, we also evaluated the contribution of non-CRP biomarkers to the MBDA score predictions. This study is intended as the first step in a comprehensive program to assess and characterize the validity and utility of the MBDA test in clinical practice.

**Significance & Innovations**

- Quantitative disease activity assessment can improve rheumatoid arthritis (RA) patient outcomes, and a multibiomarker disease activity (MBDA) score could provide an objective, consistent, and biologically rich measure of RA disease activity.
- We validated a prespecified MBDA score as a measure of RA disease activity, and showed that it was significantly associated with the Disease Activity Score in 28 joints using the C-reactive protein level and other clinical disease activity measures in both seronegative and seropositive patients.
- We also found that the MBDA score tracked changes in disease activity over time and that changes in the MBDA score quickly discriminated clinical responders from nonresponders.
- The MBDA score represents a novel disease activity index that may complement clinical assessment when the physician desires additional information to guide the management of RA in patients with this condition.

## MATERIALS AND METHODS

### Patients and samples

All patients and samples were independent of those studied during prior research and algorithm development efforts. Patients were selected from 3 cohorts: 1) the Index for Rheumatoid Arthritis Measurement (InFoRM) (27), a North American multicenter, longitudinal, observational study of patients with RA; 2) the Brigham and Women's Hospital Rheumatoid Arthritis Sequential Study (BRASS) registry (28); and 3) the Leiden Early Arthritis Clinic cohort (29). Patients positive for either RF and/or anti–cyclic citrullinated peptide antibodies (anti-CCP) were defined as seropositive. Patients negative for both RF and anti-CCP were defined as seronegative. Patient selection is described below; no other restrictions were applied.

#### Seropositive validation and performance

To evaluate the association of the MBDA score with the DAS28-CRP in seropositive patients, 230 patients were selected from the Leiden cohort (n = 77) (29), the BRASS registry (n = 87) (28), and the InFoRM study (n = 66) (27), with the objective of creating a uniform distribution of DAS28-CRP values. This procedure was enriched for patients with very low and very high DAS28-CRP scores, which increased the power to detect an association, increased the observed correlation and area under the receiver operating characteristic curve (AUROC), and enabled us to verify the MBDA score performance over a wide spectrum of disease activity.

#### Seronegative validation

To validate the MBDA score in seronegative patients across the range of disease activity, 141 patients were selected from InFoRM (n = 23) and BRASS (n = 118) with the same objective of creating a uniform distribution of the DAS28-CRP, as described above.

#### Seronegative performance

To characterize MBDA score performance in a more representative, real-world group of seronegative patients, 141 patients were selected from InFoRM (n = 75) and BRASS (n = 66) to be representative of each study's disease activity distribution (86 overlapping patients were included in both the validation and performance cohorts).

#### Treatment response

The association between changes in the MBDA score and clinical response to treatment was evaluated in patients in the Nested-1 study, a prospective observational study of MTX and anti-TNF treatment designed to identify treatment response biomarkers and conducted within the BRASS cohort (30). Eligibility criteria for Nested-1 were rheumatologists' diagnosis of active RA (≥6 tender joints and ≥6 swollen joints), age ≥18 years, stable therapy (no change in prednisone use [≤10 mg or less] in the last 4 weeks, either not receiving MTX or receiving MTX for at least 3 months with a stable dose for 4 weeks), no previous anti-TNF therapy use, and initiating treatment with either MTX or anti-TNF (with or without MTX). Sixty-eight patients were enrolled and followed for 6 to 12 weeks. Patients not achieving an ACR criteria for 20% improvement (ACR20) response at week 6 were assessed again at week 12. The DAS28-CRP was assessed at baseline and weeks 6 and 12 (if a 12-week visit was required). Serum samples were collected at baseline and weeks 2, 6, and 12 (if a 12-week visit was required). We studied all 45 patients who had complete clinical and sample data for baseline and a final evaluation visit (6 or 12 weeks). Patients were defined as DAS28-CRP responders if their improvement in the DAS28-CRP score was >1.2 and their absolute DAS28-CRP score was ≤3.2 at the final visit, analogous to the DAS28-ESR–based definition of a EULAR good response.

### Sample handling and assays

As part of the InFoRM study, samples were collected in BD Vacutainer SST Serum-Separation tubes, processed at the study site, and then shipped overnight and kept cold (2–8°C) using NanoCool shippers (NanoPore) to Crescendo Bioscience. For the BRASS and Leiden studies, samples were collected and stored according to the respective study protocols and then shipped frozen to Crescendo Bioscience. Upon arrival, all samples were stored at −80°C until analyzed.

The 12 biomarkers used to calculate the MBDA score were measured on the Meso Scale Discovery Multi-Array platform. Assay kits were manufactured by Crescendo Bioscience with some components manufactured under contract by Meso Scale Discovery. Assays used reagents that block RF (31). Assays were optimized to meet strict performance criteria and underwent rigorous analytical validation for precision, sensitivity, specificity, and lot-to-lot variability (32). Samples for validation and performance studies were run in Crescendo Bioscience's Clinical Laboratory Improvement Amendments–certified clinical laboratory. Samples from the Nested-1 study were analyzed in the Crescendo Bioscience development laboratory.

### MBDA algorithm and score calculation

#### Background on the development of the MBDA algorithm

In a previous article (23), we carried out a series of studies to develop the MBDA algorithm (Vectra DA). Starting with 396 candidate biomarkers, we analyzed the existing literature and samples from several cohorts to evaluate measurability, association with disease activity, and the incremental independent information contributed to multivariate models associating the biomarkers with clinical disease activity. These efforts led to the development of an algorithm that combines the levels of 12 biomarkers, i.e., EGF, VEGF-A, leptin, IL-6, SAA, CRP, VCAM-1, MMP-1, MMP-3, TNFRI, human cartilage glycoprotein 39 (YKL-40), and resistin, into a composite MBDA score. Results obtained during algorithm verification indicated that the MBDA score was significantly associated with the DAS28-CRP (33).

#### MBDA score calculation

**Figure 1:**
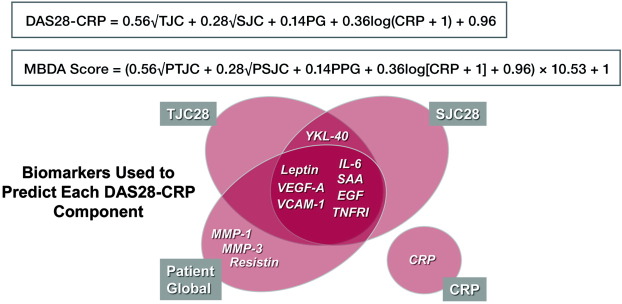
Biomarkers used to estimate each component of the Disease Activity Score in 28 joints using the C-reactive protein level (DAS28-CRP) in the multibiomarker disease activity (MBDA) algorithm. The algorithm uses different subsets of biomarkers and/or different weightings to predict each component of the DAS28-CRP, including the tender joint count (TJC), swollen joint count (SJC), patient global assessment (PG), and CRP level. The resulting mathematical relationship between the DAS28-CRP and MBDA score is: MBDA = (DAS28-CRP) × 10.53 + 1. PTJC = predicted TJC; PSJC = predicted SJC; PPG = predicted PG; TJC28 = 28-joint TJC; SJC28 = 28-joint SJC; VEGF-A = vascular endothelial growth factor A; VCAM-1 = vascular cell adhesion molecule 1; IL-6 = interleukin-6; SAA1 = serum amyloid A1; EGF = epidermal growth factor; TNFRI = tumor necrosis factor receptor type I; MMP-1 = matrix metalloproteinase 1.

Biomarker-based predictions of 28-joint tender joint count (TJC28), 28-joint swollen joint count (SJC28), and patient global assessment were made separately from subsets of the 11 non-CRP biomarkers and combined with CRP in an equation analogous to the DAS28-CRP formula (Figure 1). The formulas used to predict TJC28, SJC28, and patient global assessment can be found in Supplementary Table 1 (available in the online version of this article at http://onlinelibrary.wiley.com/journal/10.1002/(ISSN)2151-4658). Resulting MBDA scores are within the range 1–100 and are rounded to the nearest integer. The MBDA thresholds for disease activity categories were determined by translating the DAS28-CRP thresholds (34) to the corresponding MBDA scores based on the linear relationship between the DAS28-CRP and the MBDA (Figure 1).

### Statistical methods

The R software package was used for data analysis. Except where noted, a *P* value less than 0.05 was considered statistically significant.

#### Validation

The performance of the MBDA score compared to clinical disease activity measures was assessed in seropositive and seronegative groups and in the Nested-1 study separately. The primary validation outcome was the AUROC, a measure of discrimination, to classify patients according to the DAS28-CRP–based definitions of low versus moderate/high disease activity using a cut point of 2.67. This threshold is considered equivalent to a DAS28-ESR threshold of 3.2 (34).

#### Performance

Pearson's correlations between the MBDA score and the DAS28-CRP were evaluated in secondary analysis. Other analyses were exploratory. The AUROC was also used to test the association between the MBDA score and the DAS28-CRP in subgroups defined by sex, age (<65 versus ≥65 years), body mass index (BMI; >25 versus ≤25 kg/m^2^), or RA therapy (anti-TNF medications, MTX without biologic agents, and glucocorticoids) in seropositive and seronegative patients separately. An AUROC of 0.5 indicates a result no better than chance; a Pearson's correlation coefficient of 0 indicates no association. The agreement between disease activity categorizations (low, moderate, and high) was assessed by Cohen's kappa with squared distance weights (35).

#### Contribution of the non-CRP biomarkers

In exploratory analysis, the incremental contribution of the 11 biomarkers other than CRP in the MBDA algorithm was assessed via multivariate regression. The contributions of CRP and the MBDA score (without CRP) to the association with 2 outcomes were assessed: DAS28-CRP and DAS28-CRP with CRP removed. The CRP concentration was log-transformed for approximate normality and consistency with the DAS28-CRP and MBDA score calculations.

#### Change in MBDA versus therapy response

In the primary analysis, we evaluated whether changes in the MBDA score from baseline to the final visit (week 6 or 12) could differentiate DAS28-CRP responders from nonresponders at the final visit, using the AUROC. Secondary analyses included repeating the primary analysis for an ACR50 response at the final visit, evaluating the AUROC for MBDA change from baseline to week 2 differentiating the DAS28-CRP response and ACR50 response (36) at the final visit, and calculating the AUROC for the MBDA score differentiating low versus moderate/high DAS28-CRP.

#### Nested-1 exploratory analyses

Analyses not specified above were exploratory. Paired *t*-tests were used to evaluate the change in MBDA score from baseline. *P* values for comparing the performance of Vectra DA with CRP were computed using bootstrap resampling (37). In order to calculate the correlation between change in the MBDA score and a continuous measure of ACR response, the ACR-N was used (36, 38). This study was not adequately powered to examine the association with response in the MTX and anti-TNF treatment arms separately, so these arms were pooled.

## RESULTS

### Patient characteristics

The MBDA score was evaluated in 4 cohorts (Table 1). Across the cohorts, the median age ranged from 54–58 years, the median TJC28 ranged from 2–13, the median SJC28 ranged from 2–12, and the median DAS28-CRP ranged from 3.5–5.5. The Nested-1 cohort, used to evaluate therapy response, required active disease at study entry and had higher baseline disease activity than the other cohorts.

**Table 1:** Patient characteristics in cohorts used for multibiomarker disease activity validation, performance assessment, and treatment response evaluation*

	Seropositive validation and performance	Seronegative validation	Seronegative performance	Treatment response†
No. of patients	230	141	141	45
Women, %	77	82	79	84
Age, years	58 (48–66)	57 (45–65)	58 (49–65)	54 (39–64)
RF positive, %	93	0	0	73
Anti-CCP positive, %	88	0	0	N/A
TJC28	5 (0–18)	2 (0–10)	2 (0–8)	13 (10–17)
SJC28	4 (0–12)	6 (1–14)	2 (0–8)	12 (9–17)
CRP level, mg/liter	7 (3–17)	3 (0.9–9)	3 (1–9)	12 (3–30)
PG, mean (IQR)	42 (19–65)	39 (15–60)	40 (15–60)	56 (40–70)
DAS28-CRP	4.1 (2.3–5.8)	3.7 (2.4–4.9)	3.5 (2.4–4.7)	5.5 (4.9–6.4)

* Values are the median (interquartile range [IQR]) unless otherwise indicated. RF = rheumatoid factor; anti-CCP = anti–cyclic citrullinated peptide antibodies; N/A = not available; TJC28 = 28-joint tender joint count; SJC28 = 28-joint swollen joint count; CRP = C-reactive protein; PG = patient global assessment; DAS28 = Disease Activity Score in 28 joints.

†Baseline characteristics from the Nested-1 study.

### MBDA score validation and performance

#### Association of the MBDA score with clinical disease activity in RA patients

In the primary validation analysis, the MBDA score was significantly associated with the DAS28-CRP in both seropositive and seronegative patients (*P* < 0.001). Performance using the AUROC for classifying patients into low versus moderate to high disease activity was 0.77 and 0.70, and correlation with the DAS28-CRP was 0.56 and 0.43, in seropositive and seronegative patients, respectively. Results for combined seronegative and seropositive performance cohorts were similar (AUROC 0.76, Pearson's correlation = 0.57). The MBDA score categorized disease activity similarly to the DAS28-CRP (see Supplementary Table 2, available in the online version of this article at http://onlinelibrary.wiley.com/journal/10.1002/(ISSN)2151-4658) and was also significantly correlated with the CDAI, SDAI, and RAPID3 (Table 2). Similarly, across all visits in the Nested-1 study, the MBDA score was associated with the DAS28-CRP, with an AUROC of 0.71 (*P* = 0.01) for low versus moderate/high disease activity and an AUROC of 0.86 (*P* < 0.001) using the median DAS28-CRP as the threshold. For subgroups of seropositive and seronegative patients based on age, sex, BMI, and RA therapy, the MBDA score was significantly associated with the DAS28-CRP (*P* < 0.05) in all but the 2 smallest subgroups of patients (seronegative men: n = 26; *P* = 0.84 and seronegative RA patients treated with anti-TNF medications: n = 28; *P* = 0.06).

**Table 2:** Cross-sectional correlations with additional clinical disease activity measures for the MBDA score and CRP*

Biomarker measure	Clinical measure	Pearson's correlation	*P*	N
Seropositive validation				
MBDA score	SDAI	0.55	< 0.001	148
MBDA score	CDAI	0.48	< 0.001	148
CRP	CDAI	0.44	< 0.001	148
MBDA score	RAPID3	0.47	< 0.001	92
CRP	RAPID3	0.37	< 0.001	92
Seronegative performance				
MBDA score	SDAI	0.29	< 0.001	139
MBDA score	CDAI	0.21	0.02	139
CRP	CDAI	0.20	0.02	139
MBDA score	RAPID3	0.26	0.003	127
CRP	RAPID3	0.26	0.003	127

* C-reactive protein (CRP) was log-transformed prior to analysis because Pearson's correlation assumes normally distributed data. MBDA = multibiomarker disease activity; SDAI = Simplified Disease Activity Index; CDAI = Clinical Disease Activity Index; RAPID3 = Routine Assessment of Patient Index Data 3.

The MBDA thresholds for low/moderate and moderate/high disease activity were determined to be 29 and 44, respectively, calculated from the DAS28-CRP thresholds of 2.7 and 4.1, respectively (equivalent to DAS28 thresholds of 3.2 and 5.1, respectively) (34). Subjects categorized as having low, moderate, and high disease activity by the MBDA score had progressively higher levels of all of the individual components of the DAS28-CRP (Table 3).

**Table 3:** Clinical characteristics of patients based on multibiomarker disease activity score classification of disease activity in validation studies*

	N	TJC28, mean	SJC28, mean	PG, mean	CRP level, median mg/liter	DAS28-CRP, mean
Seropositive validation						
Low: ≤29	51	3.9	3.0	29	1.4	2.65
Moderate: >29 to 44	66	8.3	5.9	42	4.9	3.92
High: >44	113	12.0	9.1	48	17.0	5.01
Seronegative validation						
Low: ≤29	54	3.6	5.7	27	0.7	2.81
Moderate: >29 to 44	46	5.4	7.2	41	3.9	3.73
High: >44	41	9.8	11.0	52	15.0	4.98

* TJC28 = 28-joint tender joint count; SJC28 = 28-joint swollen joint count; PG = patient global assessment; CRP = C-reactive protein; DAS28 = Disease Activity Score in 28 joints.

#### Contribution of biomarkers other than CRP to the association between MBDA score and DAS28-CRP in seropositive patients

Because the MBDA score includes CRP, itself a component of the DAS28-CRP, we used multivariate regression analysis to assess whether the 11 other biomarkers in the MBDA score made a significant additional contribution to the association with the DAS28-CRP. Therefore, we assessed whether the MBDA score (without CRP) and CRP independently contributed to the prediction of the overall DAS28-CRP. We also specifically assessed their contribution to predicting the components of the DAS28-CRP other than CRP itself, by repeating the analysis for prediction of the DAS28-CRP with CRP removed. In multivariate regression analysis in seropositive patients, both the MBDA score (without CRP) and CRP were independent predictors of the overall DAS28-CRP (*P* < 0.001), but only the MBDA score (without CRP) was an independent predictor of DAS28-CRP with CRP removed (*P* < 0.001 for the MBDA score without CRP and *P* = 0.22 for CRP). In seronegative patients, however, CRP, but not the MBDA score (without CRP), was an independent predictor of the overall DAS28-CRP (*P* < 0.001 and *P* = 0.37, respectively), and neither CRP nor the MBDA score (without CRP) contributed significantly to the prediction of DAS28-CRP with CRP removed (*P* = 0.09 and *P* = 0.37, respectively).

### MBDA score and therapy response in the Nested-1 cohort

#### Association of changes in the MBDA score with changes in clinically-assessed disease activity

**Figure 2:**
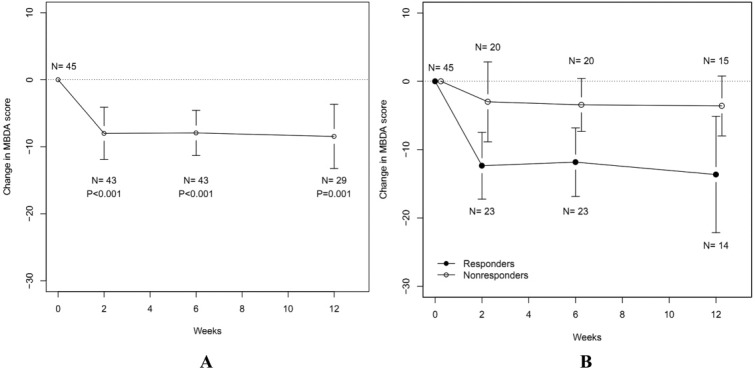
Mean changes from baseline in the multibiomarker disease activity (MBDA) score. A, Change in the MBDA score in all patients. B, Change in the MBDA score in Disease Activity Score in 28 joints using the C-reactive protein level (DAS28-CRP) responders vs. nonresponders after treatment initiation in the Nested-1 study. Error bars show the 95% confidence interval (95% CI) of mean changes in the MBDA score. *P* values in A are for the *t*-test of change from baseline. At the final visit (6 or 12 weeks), mean changes from baseline in the MBDA score were −8.4 (95% CI −4.6, −12.2) for all patients (n = 45), −13.9 (95% CI −19.7, −8.1) for DAS28-CRP responders (n = 24), and −2.1 (95% CI −5.7, 1.4) for nonresponders (n = 21). Similar results were observed for patients with complete sample data, i.e., those with MBDA scores available at 0, 2, 6, and 12 weeks (n = 27). A DAS28-CRP response was defined as a decrease in the DAS28-CRP of ≥1.2 units and an ending DAS28-CRP score of <3.2 at the last study visit.

The mean MBDA score at baseline was 48 (n = 45; 95% confidence interval 42, 54). MBDA scores dropped significantly from baseline to weeks 2, 6, and 12 (*P* ≤ 0.001), with most of the reduction occurring by week 2 (Figure 2A). Changes in MBDA scores from baseline to the final visit were significantly correlated with the corresponding changes in the DAS28-CRP (Spearman's ρ = 0.51, *P* < 0.001) and final ACR-N scores (Spearman's ρ = 0.45, *P* = 0.002).

#### Discrimination of changes in the MBDA score in clinical responders versus nonresponders

Of the 45 patients, 24 experienced a DAS28-CRP response (improvement in the DAS28-CRP score of >1.2 and in the absolute DAS28-CRP score of <3.2 at the final visit). The changes in the MBDA score in DAS28-CRP responders and nonresponders are shown in Figure 2B. In the primary analysis, the change in MBDA score from baseline to the final visit discriminated DAS28-CRP response with an AUROC of 0.77 (*P* = 0.002) (Table 4). The change in MBDA score from baseline to week 2 was also associated with DAS28-CRP response at the final visit, with an AUROC of 0.72 (*P* = 0.02). Change in the MBDA score had stronger observed associations with DAS28-CRP response than did the change in CRP, although these differences were not statistically significant.

**Table 4:** Association of changes in MBDA score and CRP with treatment-induced changes in clinical disease activity in the Nested-1 study*

Clinical outcome at final visit†	ΔMBDA score	ΔCRP‡
Correlation to change in clinical disease activity, Spearman's ρ		
Baseline to final visit†		
ΔDAS28-CRP	0.51 (*P* < 0.001)	0.43 (*P* = 0.004)
ACR-N	0.45 (*P* = 0.002)	0.33 (*P* = 0.027)
Discrimination of clinically-defined response, AUROC		
Baseline to final visit†		
DAS28-CRP response	0.77 (*P* = 0.002)	0.68 (*P* = 0.03)
ACR50 response	0.69 (*P* = 0.03)	0.59 (*P* = 0.30)
Baseline to week 2		
DAS28-CRP response	0.72 (*P* = 0.02)	0.69 (*P* = 0.03)
ACR50 response	0.65 (*P* = 0.11)	0.51 (*P* = 0.91)

* MBDA = multibiomarker disease activity; CRP = C-reactive protein; DAS28 = Disease Activity Score in 28 joints; ACR-N = American College of Rheumatology N; AUROC = area under the receiver operating characteristic curve; ACR50 = ACR criteria for 50% improvement.

† The final visit for each patient corresponds to week 6 or 12 (last available).

‡ Percent change in CRP was used for AUROC; change in log(CRP) was used for correlation analyses.

#### Comparison with CRP for discriminating ACR responses

We also compared the MBDA score to CRP for discrimination of ACR responses, which are less influenced by CRP than DAS28-CRP responses. The AUROC for change in the MBDA score at the final study visit differentiating ACR50 responders was greater than that for change in CRP (*P* = 0.04). Change in the MBDA score at week 2 was also more strongly associated with an ACR50 response than was the change in CRP (*P* = 0.007). The correlation of change in the MBDA score at the final study visit with the ACR-N was greater than that with CRP (Spearman's ρ = 0.45 versus 0.33), although this difference was not statistically significant.

## DISCUSSION

These results demonstrate a significant association between the MBDA score and the DAS28-CRP in heterogeneous groups of RA patients with diversity in autoantibody status, disease activity, and RA therapy receiving care in multiple clinical centers. The MBDA score was able to consistently distinguish patients in different categories of clinical disease activity. Our results provide evidence of criterion validity for the MBDA test by demonstrating a significant association with the DAS28-CRP and other validated disease activity measures.

As a continuous score, the MBDA score was correlated with the DAS28-CRP with coefficients of 0.56 and 0.43 in seropositive and seronegative patients, respectively. In comparison, correlations to the DAS28-ESR and DAS28-CRP reported for other clinical measures range from 0.43–0.70 for the RAPID3 and from 0.51–0.54 for the multidimensional and modified Health Assessment Questionnaires (39–41). Correlations of the SDAI and CDAI with the DAS28-CRP tend to be greater (range 0.80–0.93) (10, 11), since these 3 measures have many components in common. The MBDA score emphasizes the activity of underlying biologic pathways rather than external signs and symptoms and should therefore provide information that is different from, and complementary to, clinical assessment. Studies are underway to assess the utility of the MBDA score when used in conjunction with clinical assessment and to compare cross-sectional and longitudinal relationships of different disease activity measures (including the MBDA score) to various RA outcomes, including imaging-based (e.g., magnetic resonance imaging [MRI], ultrasound, radiographs) assessments of joint inflammation and damage progression.

In addition to criterion validity, results from the Nested-1 analysis provide evidence of the discriminant validity of the MBDA score by demonstrating that changes in the score were associated with both the DAS28-CRP and ACR50 treatment response for patients receiving MTX or anti-TNF therapy. MBDA scores declined more in clinical responders than in nonresponders, with significant changes detectable as early as 2 weeks after therapy initiation. Our finding that an early drop in MBDA score was indicative of subsequent DAS28-CRP response is intriguing and should be evaluated in larger studies to determine whether the MBDA score might allow earlier evaluation of treatment efficacy. Taken together, these findings suggest that biomarkers may help assess treatment response, even shortly after treatment initiation, in both clinical trials and clinical practice.

The face validity of the MBDA test is supported by the relationship of the component biomarkers to biologic mechanisms relevant to RA disease activity. Despite the fact that biomarker selection for the test was based on statistical contribution to estimation of disease activity, the MBDA biomarkers represent critical cytokine signaling pathways in RA (IL-6, TNFRI) as well as hallmark RA disease processes, including angiogenesis and tissue remodeling (VEGF-A, EGF), cell recruitment and invasion (VCAM-1), cartilage remodeling (MMPs), and elevated acute-phase response (CRP, SAA). Associations between many of these biomarkers and disease activity have previously been reported (42–48).

Content validity requires comprehensive evaluation of all relevant facets of disease activity (24, 25). Since the MBDA score does not include physical evaluation and is intended to complement rather than replace clinical assessment, content validity is both limited and less relevant. While the MBDA score does not reflect signs and symptoms, the 12 biomarkers are functionally diverse and more biologically comprehensive than current laboratory tests and clinical tools.

The diverse biology underlying the MBDA biomarkers is also consistent with the hypothesis that integration of information from multiple pathways can enhance disease activity assessment. To explore this hypothesis, we evaluated the contribution of non-CRP biomarkers to the MBDA score and prediction of the DAS28-CRP. Our results verified that the MBDA score without CRP was an independent predictor of the DAS28-CRP in seropositive patients, indicating that the non-CRP biomarkers provide additional information on disease activity in these patients. Characterization of the relationship between MBDA score and outcomes not themselves based on CRP, such as joint damage progression and disability, is currently underway and will offer a more meaningful way to assess the value of the MBDA score above and beyond CRP.

The patients examined in the validation studies were diverse in terms of geographic origin and disease characteristics, and they were not selected except for their clinical disease activity levels. Consequently, the study results indicate that the MBDA score is a valid measure of disease activity in representative current RA patients, including a variety of therapies and comorbidities. However, since emerging therapies for RA may have novel effects on biomarkers, it will be important to study the performance of the score in patients treated with drugs with new mechanisms of action. In addition, since some comorbidities can affect biomarker levels, it is also important to investigate whether they affect the relationship between the MBDA score and clinical disease activity. No significant effects on the MBDA score have been found for several comorbidities commonly found in patients with RA (49), but further studies are warranted.

Demonstration of the criterion, discriminant, and face validity of the MBDA score is an important step in the full qualification of this novel disease activity measure. These findings support the use of the MBDA score as an objective measure of disease activity that reflects the diverse underlying biology of RA. Ongoing and future research on the MBDA score will include evaluating its relationship to imaging-based assessment of inflammation (ultrasound and MRI) and its construct validity by assessing its ability to predict long-term outcomes such as RA flare, joint damage progression, and disability. Ultimately, the clinical utility of the MBDA test may be best determined by prospective studies that evaluate whether patient outcomes are improved by the use of the test as an adjunct to clinical assessment.

## AUTHOR CONTRIBUTIONS

All authors were involved in drafting the article or revising it critically for important intellectual content, and all authors approved the final version to be published. Dr. Curtis had full access to all of the data in the study and takes responsibility for the integrity of the data and the accuracy of the data analysis.

**Study conception and design.** Curtis, Huizinga, Haney, Shen, Cavet, Centola, Hesterberg, Chernoff, Ford, Fleischmann, Keystone, Weinblatt.

**Acquisition of data.** Curtis, van der Helm-van Mil, Knevel, Huizinga, Hesterberg, Shadick, Hamburger, Fleischmann, Keystone, Weinblatt.

**Analysis and interpretation of data.** Curtis, Huizinga, Haney, Shen, Ramanujan, Cavet, Ford, Shadick, Fleischmann, Weinblatt.

## ROLE OF THE STUDY SPONSOR

Biogen Idec and Crescendo Bioscience provided partial support for the Brigham and Women's Hospital Rheumatoid Arthritis Sequential Study. Crescendo Bioscience designed and executed the Index for Rheumatoid Arthritis Measurement study, was responsible for biomarker data collection and analysis, and contributed to writing of the manuscript. Publication of the manuscript was subject to approval by all the authors, including those employed by these sponsors, but not otherwise contingent on the sponsors' approval.
