# Supplementary material for: Validation of a novel multibiomarker test to assess rheumatoid arthritis disease activity
Source: Arthritis Care Res (Hoboken). 2012 Nov 28;64(12):1794–803. doi: 10.1002/acr.21767 (PMC3508159; doi:10.1002/acr.21767)
Supplement: Supplementary file 1 [file acr0064-1794-SD1.doc]

**Supplementary Table 1. Formulas used to estimate tender joint count, swollen joint count, and patient global assessment**

| **PTJC** | -26.72+3.243 *[YKL-40]^1/10^-11.97*[EGF]^1/10^+15.72*[IL-6]^1/10^+0.4594*[Leptin]^1/10^ +3.881*[SAA]^1/10^+0.7388*[TNF-RI]^1/10^-0.2557*[VCAM-1]^1/10^+0.7003*[VEGF-A]^1/10^ |
| --- | --- |
| **PSJC** | -26.63+3.232*[YKL-40]^1/10^-11.93*[EGF]^1/10^+15.67*[IL-6]^1/10^+0.4578*[Leptin]^1/10^ +3.868*[SAA]^1/10^+0.7363*[TNF-RI]^1/10^-0.2548*[VCAM-1]^1/10^+0.6979*[VEGF-A]^1/10^ |
| **PPG score** | -13.489+5.474*[IL-6]^1/10^+0.486*[SAA]^1/10^+2.246*[MMP-1]^1/10^+1.684*[Leptin]^1/10^+ 4.14*[TNF-RI]^1/10^+2.292*[VEGF-A]^1/10^-1.898*[EGF]^1/10^+0.028*[MMP-3]^1/10^-2.892* [VCAM-1]^1/10^-0.506*[Resistin]^1/10^ |

All biomarker concentrations are in pg/ml.

EGF = epidermal growth factor; IL-6 = interleukin-6; MMP = matrix metalloproteinase; PPG = predicted patient global; PSJC = predicted swollen joint count; PTJC = predicted tender joint count; SAA = serum amyloid A; TNF-R1 = tumor necrosis factor receptor superfamily member 1A ; VCAM-1 = vascular cell adhesion molecule 1; VEGF-A = vascular endothelial growth factor-A; YKL-40 = human cartilage glycoprotein 39.

**Supplementary Table 2. Agreement between disease activity categorizations based on the MBDA score and on DAS28-CRP**

| **Cohort** | **Kappa** | ***P* Value** |
| --- | --- | --- |
| Seropositive Validation | 0.44 | <0.001 |
| Seronegative Performance | 0.33 | <0.001 |

The categorization of patients into low, moderate and high disease activity based on the MBDA score was compared to that based on DAS28-CRP.
